# Distribution and Expression of Vimentin and Desmin in Broiler *Pectoralis major* Affected by the Growth-Related Muscular Abnormalities

**DOI:** 10.3389/fphys.2019.01581

**Published:** 2020-01-17

**Authors:** Francesca Soglia, Maurizio Mazzoni, Martina Zappaterra, Mattia Di Nunzio, Elena Babini, Martina Bordini, Federico Sirri, Paolo Clavenzani, Roberta Davoli, Massimiliano Petracci

**Affiliations:** ^1^Department of Agricultural and Food Sciences, Alma Mater Studiorum – University of Bologna, Bologna, Italy; ^2^Department of Veterinary Medical Sciences, Alma Mater Studiorum – University of Bologna, Bologna, Italy

**Keywords:** vimentin, desmin, white striping, wooden breast, spaghetti meat

## Abstract

Desmin (DES) and Vimentin (VIM) exert an essential role in maintaining muscle cytoarchitecture and since are considered reliable markers for muscle regeneration, their expression has been extensively investigated in dystrophic muscles. Thus, exhibiting features similar to those of human dystrophic muscles, the present study aimed at assessing the distribution of VIM and DES proteins and the expression of the corresponding genes in *Pectoralis major* muscles affected by white striping (WS), wooden breast (WB), and spaghetti meat (SM) abnormalities as well as in those having macroscopically normal appearance (NORM). For this purpose, 20 *Pectoralis major* muscles (5/group) were collected from the same flock of fast-growing broilers to perform immunohistochemistry, immunoblotting and gene expression. Immunohistochemical analyses showed an increased number of fibers immunoreactive to both VIM and DES in WS and WB, while only a few immunoreactive fibers were observed in NORM. Concerning the protein level, if compared with NORM, a 55% increase in VIM content was found in WB affected cases (*P* < 0.05) thus suggesting the development of intense regenerative processes in an early-stage within these muscles. The significantly higher amount of DES (+53%) found in WS might be attributed to a progression of the regenerative processes that require its synthesis to preserve the structural organization of the developing fibers. On the other hand, significantly lower VIM and DES contents were found in SM. About gene expression, *VIM* mRNA levels gradually increased from the NORM to the SM group, with significantly higher gene expressions in WB and SM samples compared to the NORM group (*P* = 0.009 for WB vs. NORM and *P* = 0.004 for SM vs. NORM). Similarly, the expression of *DES* gene showed an increase from the NORM to WB group (*P* = 0.05). Overall, the findings of the present study suggest that intense regenerative processes take place in both WB and WS muscles although a different progression of regeneration might be hypothesized. On the other hand, the lack of correspondence between *VIM* gene expression and its protein product observed in SM suggests that VIM may also exert a role in the development of the SM phenotype.

## Introduction

In the past few years, several studies have been carried out in order to investigate the underpinning factors involved in the development of the muscular growth-related abnormalities [White Striping (WS), Wooden Breast (WB), and Spaghetti Meat (SM)] that are currently affecting the *Pectoralis major* (*P. major*) muscles in modern fast-growing broilers (Petracci et al., 2019). Although previous studies deeply investigated the role of genetics (Mutryn et al., 2015; Alnahhas et al., 2016; Zambonelli et al., 2016; Pampouille et al., 2018; Papah et al., 2018), the exact etiology and the chronology of events leading to the development of these conditions are not fully understood. The microscopic examinations of the *P. major* affected by the aforementioned abnormalities evidence a profoundly altered muscular architecture with fiber degeneration up to necrosis, and concomitant occasional regenerative processes (Velleman, 2019). Besides, the skeletal muscle shows a proliferation of poorly organized connective tissue (fibrosis) and increased fat deposition (lipidosis) (Soglia et al., 2019).

Ultrastructural studies (Granger and Lazarides, 1979; Tokuyasu et al., 1985) demonstrated that, by constituting a three-dimensional scaffold around the Z-disk, vimentin (VIM) and desmin (DES) play a relevant role in maintaining sarcomere cytoarchitecture. DES is an important component of the cytoskeleton of striated muscles (Lazarides, 1980) located at the periphery of Z-disks where it is arranged in a honeycomb-like structure within the Z plane of the myofibers and is involved in the connection of neighboring Z-disks (Granger and Lazarides, 1979). On the other hand, VIM is mainly located in cells of mesenchymal origin including myoblasts (Price and Sanger, 1982) and co-localize with desmin (Granger and Lazarides, 1979). Although DES and VIM coexist during early myogenesis, the last is gradually reduced as the development of myotubes proceeds (Tokuyasu et al., 1985). In detail, in early myogenesis and immature myotubes, VIM and DES are found as longitudinally arranged filaments and randomly distributed cytoplasmic strands, respectively (Gard and Lazarides, 1980). Their peculiar arrangements in muscle tissue might play an essential role in maintaining the cylindrical form of developing myotubes and allowing to parallelly align the myofibers (Tokuyasu et al., 1985). In particular, it was demonstrated that as the newly synthesized DES filaments converge and replace the pre-existing VIM-based network, DES distribution within the fibers reflects that of VIM (Bennett et al., 1979; Granger and Lazarides, 1979; Cary and Klymkowsky, 1994). Subsequently, a substantial re-organization of the intermediate filaments scaffolding takes place and involves an evolution from a longitudinally to a transversely arranged system. Concurrently, the composition of the intermediate filaments is profoundly changed: VIM synthesis is reduced whereas DES arises as the main component of mature muscle cells (Bennett et al., 1979; Granger and Lazarides, 1979).

In this context, because of their importance for maintaining muscle cytoarchitecture, the expression and distribution of VIM and DES have been previously investigated in humans affected by different neuromuscular and myopathic disorders including polymyositis, central core myopathy, nemaline myopathy and Duchenne muscular dystrophy (Gallanti et al., 1992; Banwell, 2001), but also in mdx mice (Holland et al., 2015), pigs used as a model for studying Duchenne muscular dystrophy (Fröhlich et al., 2016) and Golden retrievers affected by muscular dystrophy (Cooper et al., 1988). As a consequence, an increased abundance of VIM and DES is undoubtedly considered as an evidence of muscular dystrophy and can be considered a reliable marker for the regenerative processes taking place within the muscle tissue (Bornemann and Schmalbruch, 1992; Gallanti et al., 1992; Fröhlich et al., 2016). Within this context, since the microscopic examinations carried out on *P. major* muscles of broiler chickens affected by WS, WB, and SM abnormalities evidenced the occurrence of occasional regenerative processes (Baldi et al., 2018; Velleman, 2019), it seems reasonable to hypothesize that the expression and distribution of the intermediate filaments VIM and DES might be involved in the occurrence of these conditions. Furthermore, evident signs of regeneration are found not only within the WS, WB, and SM affected muscles but even within those cases exhibiting a macroscopically normal appearance (Soglia et al., 2019). Thus, the present study aimed at assessing the distribution of VIM and DES as well as the expression of the corresponding genes within the *P. major* muscle samples exhibiting either a macroscopically normal appearance or showing WS, WB, and SM phenotypes in order to evaluate the possible implication of these two intermediate filaments components in the occurrence of the aforementioned conditions in fast-growing chickens.

## Materials and Methods

Twenty *P. major* muscles (5 muscles/group) were collected at 3 h *post-mortem* from the same flock of fast-growing broilers farmed and slaughtered under commercial conditions (45 day-old Ross 308 males slaughtered at 3.0 kg live weight). All samples, 6 × 3 × 1 cm in size, were excised from the ventral surface of the *P. major* muscle (facing the skin), excluding the most superficial 3 mm. According to the procedure described in our previous study (Soglia et al., 2017), samples collected for immunohistochemistry and protein extraction were excised from the superficial section of the cranial portion of the *P. major*, quickly frozen in isopentane (cooled with liquid nitrogen) and stored at −80°C until processing. In detail, to assess the distribution and expression of the intermediate filament proteins DES and VIM within the muscles, immunohistochemical analyses SDS-PAGE followed by immunoblotting were carried out. Further samples of the same *P. major* were excised from the same position, immediately frozen in liquid nitrogen and then stored in a deep freezer until RNA extraction. RNA was extracted individually from the 22 gathered samples (2 *P. major* muscles belonging to the slow-growing genotype and the 20 fast-growing ones) and the expression of the genes encoding for *VIM* and DES was assessed by RT-qPCR. According to their macroscopic features the *P. major* were classified as normal (NORM), WS, WB, and SM following the criteria adopted in our previous studies (Soglia et al., 2016; Baldi et al., 2018). Only severe cases were sampled and, to avoid eventual interference, the muscles concurrently affected by more than one defect (i.e., combined WS/WB and WS/SM) were discarded. In addition, two *P. major* samples were collected from slow-growing chickens farmed and slaughtered under commercial conditions (160 day-old Leghorn cocks slaughtered at 2.5 kg of live weight). The findings obtained for these two slow-growing birds were compared with those found for the NORM muscles belonging to the fast-growing group. In this way, we can obtain both a comparison with a control group belonging to the fast-growing genotype (NORM vs. the *P. major* with abnormalities) and a further comparison between the NORM fast-growing group with its slow-growing and not selected for meat production purpose counterpart. All the farming and slaughter procedures performed in this study were in accomplishment with the Italian legislation, Legislative decree of the 4^th^ of March 2014 No. 26, article 2 point F, and did not require further specific authorization. Moreover, all farming procedures followed the Council Directive 98/58/EC concerning the protection of animals kept for farming purposes, Council Directive 2008/120/EC and Council Directive 43/2007/EC laying down minimum rules for the protection of chickens kept for meat production. Animal transport was performed according to Council Regulation (EC) No. 1/2005, slaughter was performed in accomplishment with the Council Regulation (EC) No. 1099/2009 on the protection of animals at the time of killing and under the control of the Veterinary Service from the Italian Ministry of Health, as indicated in the Regulation (EU) 2017/625 of the European Parliament. The samples used for the present study were gathered from carcasses intended for meat consumption. The research did not involve any experiment on animals and for this reason, no ethics approval was necessary.

### Immunohistochemistry

The specimens were oriented for the transverse fibers sectioning. For each *P. major* muscle, 10 serial cross-sections (10 μm thick) were cut on a cryostat microtome at −20°C and mounted on poly-L-lysine coated glass slides (Sigma-Aldrich, St. Louis, MO, United States). For immunohistochemistry, the avidin-biotin-peroxidase complex (ABC) method was used. Briefly, the sections were rinsed in phosphate buffer saline and incubated in 5% normal goat serum (for 30 min at room temperature) to reduce the non-specific binding of the secondary antibodies. The sections were then incubated at 4°C in a humid chamber for 24 h with the monoclonal mouse antiserum anti-VIM and the polyclonal rabbit antiserum anti-DES (61013 and 10570, Progen Biotechnik GmbH, Heidelberg, Germany, respectively) both diluted 1:1000. After washing, the sections were incubated at room temperature for 1 h with the biotin-conjugated goat anti-mouse IgG and biotin-conjugated goat anti-rabbit IgG secondary antibodies, both diluted 1:200 (Vector Laboratories, Burlingame, CA, United States), and then treated with ABC (Vector elite kit, Vector Laboratories, Burlingame, CA, United States). The immune reactions were visualized through a 3,3′-diaminobenzidine (DAB) chromogen solution (Vector DAB kit, Vector Laboratories, Burlingame, CA, United States).

### SDS-PAGE Analysis

The myofibrillar and sarcoplasmic proteins were extracted following the procedure described by Liu et al. (2014) with slight modifications. Briefly, one gram (in duplicate) of frozen *P. major* muscle was homogenized by Ultra-Turrax (IKA, Germany) (30 s at 13,000 rpm, in ice) in 20 mL of cold Rigor Buffer (75 mM KCl, 10 mM KH_2_PO_4_, 2 mM MgCl_2_, 2 mM EGTA; pH 7.0) (Sigma-Aldrich, St. Louis, MO, United States). The homogenate was centrifuged for 10 min at 10,000 × *g* at 4°C and the supernatant collected (sarcoplasmic protein). The procedure was repeated and the resultant pellet, identified as the myofibrillar protein fraction, was re-suspended by homogenization in 20 mL of cold Rigor Buffer. After being quantified (Bradford, 1976) the protein concentration (both myofibrillar and sarcoplasmic proteins) of each extract was adjusted to 1.0 mg/mL and each sample was mixed 1:1 (v/v) with Sample Buffer (50 mM Tris-HCl, 8M Urea, 2M Thiourea, 75 mM DTT, 3% (v/v) SDS; pH 6.8) (Sigma-Aldrich, St. Louis, MO, United States) (Fritz et al., 1989). SDS-PAGE analysis was run, in duplicates, on 5 μg of proteins according to the procedure described by Laemmli (1970) by using 7.5% polyacrylamide hand-cast gels. A molecular weight marker (Precision Plus Standard Proteins, All Blue Prestained, Bio-Rad Laboratories, Hercules, CA, United States) was loaded into each gel to assess the molecular weight of the protein bands. Gels were run on a Bio-Rad Mini Protean II electrophoresis apparatus at constant voltage (110 V) for about 1 h. Gels were stained with Coomassie Brilliant Blue R-250 (1 g/L) containing 40% (v/v) methanol and 10% (v/v) acetic acid (Sigma-Aldrich, St. Louis, MO, United States) in distilled water and de-stained in distilled water. Gel images were acquired by using a GS-800^TM^ Calibrated Densitometer (Bio-Rad Laboratories).

### Western Blot

Myofibrillar proteins (10 μg) were loaded in 10% Mini-PROTEAN TGX Stain-Fee^TM^ Gels (Bio-Rad Laboratories), which are able to produce, after UV-induction, a stable, quantitative, and western blotting compatible protein fluorescent signal due to the reaction of the trihalocompound incorporated into gel formulations with the tryptophan residues contained in proteins. After electrophoresis (carried out in the same conditions described earlier), gels were activated by UV exposure for 5 min and protein fluorescence was acquired using a ChemiDoc^TM^ MP Imaging System (Bio-Rad Laboratories) with the Image Lab software (version 5.2.1). Proteins were then transferred onto a nitrocellulose membrane using a trans-blot turbo system (Bio-Rad Laboratories) and probed at room temperature for 60 min with the specific monoclonal mouse antiserum anti-VIM and the polyclonal rabbit antiserum anti-DES (61013 and 10570, Progen Biotechnik GmbH, Heidelberg, Germany, respectively) both diluted 1:10,000. After washing, the membranes were incubated with secondary anti-mouse and anti-rabbit antibodies for 60 min (1:15,000) (Merk Millipore, Burlington, MA, United States). Membranes were subsequently incubated at room temperature with HRP-conjugated streptavidin (Merk Millipore, Burlington, MA, United States) for 20 min. Final detection was performed with enhanced chemiluminescence (Clarity^TM^ Western ECL Substrate) Western Blotting detection kit (Bio-Rad Laboratories) and the images were acquired using the ChemiDoc^TM^ MP Imaging System (Bio-Rad Laboratories). Densitometry differences were analyzed with the Image Lab software and normalized for the total fluorescent protein signal intensity (Valli et al., 2018).

### Gene Expression

Total RNA was extracted using TRIzol^®^ reagent (15596026, Invitrogen^TM^, Thermo Fisher Scientific, Waltham, MA, United States), the quality and integrity of the RNA were both checked and RNA was retrotranscribed to complementary DNA (cDNA) using iScript^TM^ cDNA Synthesis Kit (1708891, Bio-Rad Laboratories) following manufacturer’s instructions. Primers were designed using Primer3Plus web software^1^ and the complete information of the sequences are reported in Supplementary Table 1. Since Ensembl on-line genome database reported two different transcripts for the *Gallus gallus VIM* gene (last accessed on 30^th^ of January 2019), two primer couples have been designed to quantify them. In particular, a couple of primers was designed on the nucleotide sequence common to both *VIM* isoforms, while another one was conceived to amplify a sequence specific to the longer *VIM* transcript. Gene expression was analyzed by quantitative Real-Time PCR (RT-qPCR) using the standard curve method (Pfaffl, 2004) on Rotor-Gene^TM^ 6000 (Corbett Life Science, Concorde, NSW, Australia). The samples were first used to assess the expression level of three normalizing genes already tested also in previous studies (Bagés et al., 2015; Zambonelli et al., 2016): glyceraldehyde-3-phosphate dehydrogenase (*GAPDH*), ribosomal protein L32 (*RPL32*) and tyrosine 3-monooxygenase/tryptophan 5-monooxygenase activation protein, zeta (*YWHAZ*). Primers and the used RT-qPCR conditions are reported in Supplementary Table 1. The expression levels of these 3 genes were evaluated using NormFinder (Andersen et al., 2004) and *RPL32* and *YWHAZ*, the 2 most stably expressed normalizing genes, were used as reference genes to normalize the expression of *DES* and *VIM* genes. For each gene, a couple of primers has been designed and used to obtain the amplicon for the standard curve and to quantify the gene expression. Threshold cycles obtained for the samples were converted by Rotor-Gene^TM^ 6000 in mRNA molecules/μl using for each gene the relative standard curve (Pfaffl, 2004). Samples were assayed at least in triplicate to obtain, among the repetitions, coefficients of variation below 0.2.

### Statistical Analysis

Data concerning SDS-PAGE and Western Blot analyses were analyzed by using the one-way ANOVA option of Statistica 10 (StatSoft Inc., 2014) considering the type of abnormality (NORM, WS, WB, and SM) as the main effect. The same model was furthermore applied to analyze the normalized gene expressions of the *DES* gene and the two *VIM* transcripts. Mean values were subsequently separated through the parametric Tukey-HSD test. Also, Student’s *t*-test was applied to test the differences in protein and gene expression levels between the NORM muscles belonging to the fast-growing and their slow-growing counterpart. All statistical differences were considered significant at a level of *P* ≤ 0.05. In addition, Spearman’s correlations among the gene expressions of *DES* and *VIM* transcripts were calculated on the whole dataset of 20 *P. major* samples and inside each group (NORM, WS, WB, and SM) to find gene expression patterns (Zappaterra et al., 2015). Correlations were performed using rcorr function of *Hmisc* package in R environment (R Core Team, 2018) and nominal *P* ≤ 0.05 was considered statistically significant.

## Results

### Immunohistochemistry

**FIGURE 1:**
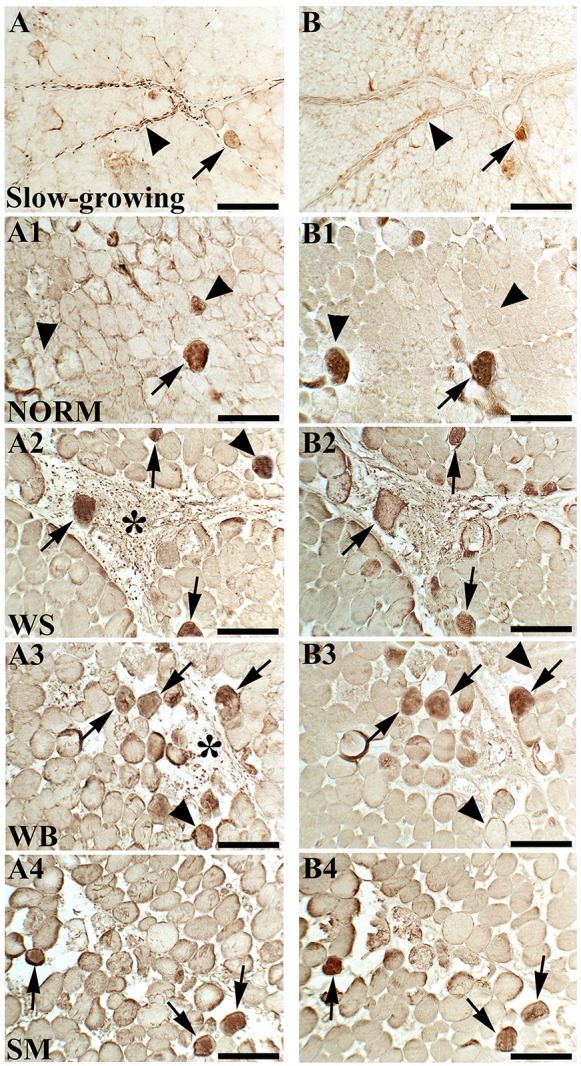
Immunoreactivity for vimentin (VIM) and desmin (DES) assessed in a slow-growing genotype **(A,B)** as well as in *Pectoralis major* muscles of fast-growing chickens exhibiting either macroscopically normal appearance (NORM; **A1,B1**) or affected by White Striping (WS; **A2,B2**), Wooden Breast (WB; **A3,B3**), and Spaghetti Meat (SM; **A4,B4**) abnormalities. Some fibers were immunoreactive to both VIM and DES (arrows) whereas other were positive to VIM but not to DES (arrowheads). Fibroblasts were positively stained to VIM (asterisks). Bars = 200 μm.

The results for immunohistochemical analyses are reported in Figure 1. Only a few fibers immunoreactive to VIM and DES were found in NORM muscles (Figures 1A1,B1). On the other hand, an increased number of fibers immunoreactive to both VIM and DES were observed in *P. major* affected by muscular abnormalities (Figures 1A2,A3,B2,B3). In detail, many positively stained fibers were found in WS and WB muscles, in which several fibroblasts exhibited a strong VIM immunoreactivity (Figures 1A2,A3,B2,B3). Similarly, some fibers showed diffuse immunoreactivity to both VIM and DES in SM affected muscles (Figures 1A4,B4). Overall, in WS, WB and SM affected muscles, most of the fibers positively stained to VIM and DES exhibited a strong immunoreactivity at the sarcolemmal and sub-sarcolemmal levels, whereas a weak or lacking reaction was found in the area corresponding to the core. Besides, some fibers were immunoreactive to VIM but not to DES (Figures 1A1–A3,B1–B3). Astonishing differences were found by comparing the results obtained for the NORM muscles belonging to fast-growing with those found for the slow-growing genotype, shown in Figures 1A,B. In detail, VIM immunoreactivity was observed within the intermyofibrillar network and, although only a few fibers were positively stained in sub-sarcolemmal position, a strong immunoreaction was found in fibroblasts (Figure 1A). Only a few fibers were positively stained to both VIM and DES and a partial reactivity to DES was observed in correspondence to the perimysial compartment (Figures 1A,B).

### SDS-PAGE and Western Blot Analyses

**FIGURE 2:**
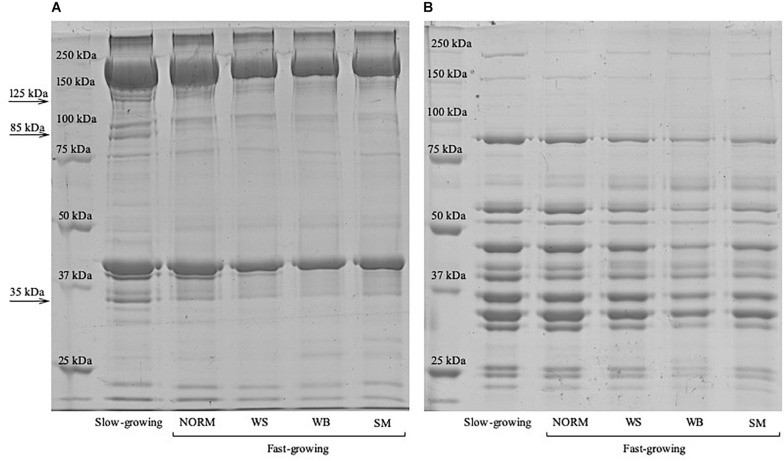
Coomassie Blue stained SDS-PAGE of the myofibrillar **(A)** and sarcoplasmic **(B)** proteins extracted from the *Pectoralis major* muscles of the slow-growing as well as from the fast-growing genotype exhibiting either macroscopically normal appearance (NORM) or affected by White Striping (WS), Wooden Breast (WB), and Spaghetti Meat (SM) abnormalities.

The electrophoretic profiles of the myofibrillar and the sarcoplasmic proteins assessed on *P. major* belonging to the fast-growing genotype (NORM, WS, WB, and SM) as well as in those muscles excised from the slow-growing strain are displayed in Figures 2A,B, respectively. Although the electrophoretic patterns did not differ among the samples belonging to fast-growing groups, relevant variations can be observed by comparing the intensity of the bands ascribed to the myofibrillar protein fraction of the fast-growing and of the slow-growing genotype. Indeed, if compared with NORM, a significant increase (*P* ≤ 0.001) in the optical densities of the bands having a molecular weight of 35, 85 and 125 kDa were found in slow-growing chickens: +57, +54, and +26%, respectively.

**FIGURE 3:**
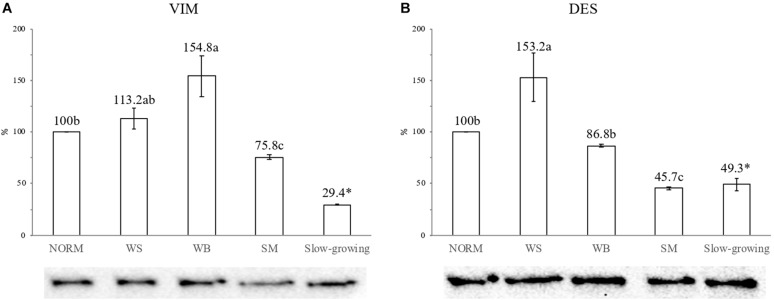
Effect of the occurrence of White Striping (WS), Wooden Breast (WB), and Spaghetti Meat (SM) abnormalities on the content of VIM **(A)** and DES **(B)** proteins in the *Pectoralis major* muscles of the fast-growing genotype assessed through Western Blot analyses. The results were analyzed by One-way ANOVA and expressed as%, considering as 100% the intensity of the band assigned to VIM and DES in normal (NORM) muscles. Error bars indicate standard deviations. a–c = Mean values followed by different superscript letters significantly differ among the fast-growing groups (*P* ≤ 0.05). On the other hand, the comparison between fast-growing and slow-growing genotype was carried out by using Student’s *t*-test. ^∗^ = Mean value for the slow-growing genotype followed by ^∗^ significantly differ from that assessed in NORM muscles belonging to the fast-growing group (*P* ≤ 0.05).

Figure 3 shows the findings concerning the expression level of VIM and DES assessed through Western Blot analyses. Concerning VIM (Figure 3A), if compared with NORM, a 55% increase in the expression level of the protein was found in WB affected muscles (100 vs. 155%; *P* ≤ 0.05) whereas WS exhibited intermediate values (113%). As for DES (Figure 3B), a 53% increased expression of this protein was observed in WS muscles whereas the content measured in WB did not differ (*P* > 0.05) from that of the NORM group. In addition, a significant reduction (*P* ≤ 0.05) in the expression level of both VIM and DES (−24 and −54%, respectively) was observed in SM.

Remarkable differences were found by comparing the findings concerning the expression level of VIM and DES in NORM fast-growing muscles with those excised from the slow-growing genotype. Indeed, if compared with their fast-growing counterpart, a significant reduction (*P* ≤ 0.001) in the content of both VIM and DES was found in slow-growing samples (−71 and −51%, respectively).

### Gene Expression

**FIGURE 4:**
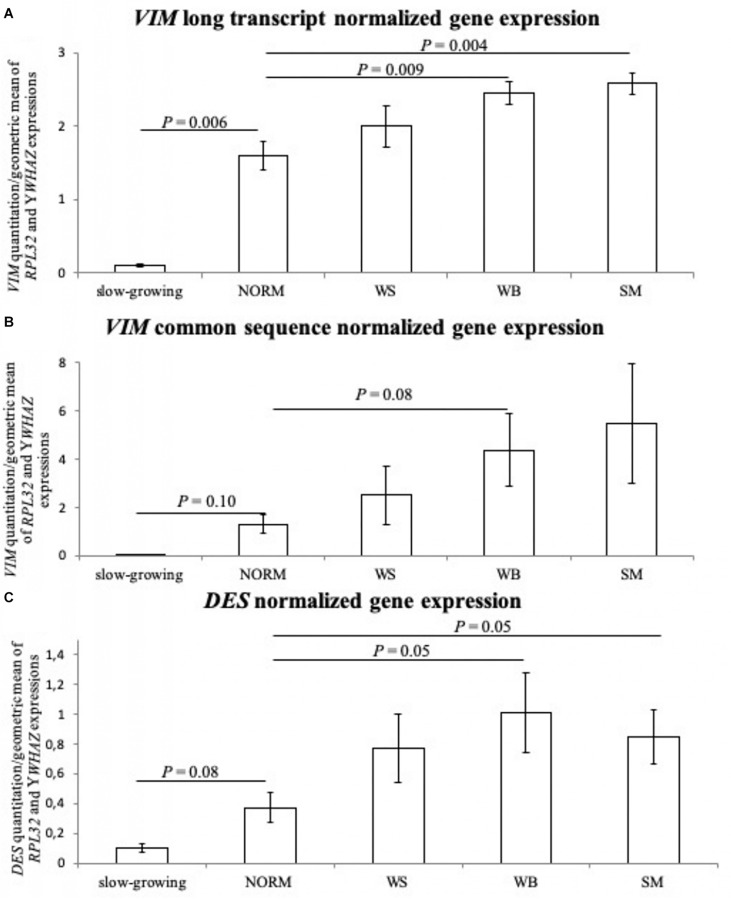
The normalized expression values observed for the tested groups: normal (NORM), White-Striping (WS), Wooden Breast (WB), and Spaghetti Meat (SM) samples. Furthermore, the normalized gene expression levels were also reported for the slow-growing samples, and their values were used to compare the gene expressions measured in NORM group. Panel **(A)** shows the normalized expression levels obtained with the primer couple specific for *Vimentin* (*VIM*) long-isoform; **(B)** reports the normalized expression levels obtained with the primers designed on *Vimentin* (*VIM*) sequence common to both gene isoform; **(C)** shows the normalized expression levels of *Desmin* (*DES*) gene. The measured standard errors are graphically represented by error bars, and only the significant *P*-values (*P* ≤ 0.05) and trends toward significance (*P* ≤ 0.10) are reported for the comparisons between group gene expressions.

The normalized gene expression levels of the two *VIM* sequences and *DES* gene are reported in Table 1 and Figure 4. Both *VIM* gene sequences showed differences in their expressions between groups (*P* ≤ 0.05 for the group effect, in Table 1) with transcription levels progressively increasing when passing from the NORM to the SM (Figures 4A,B). In particular, the normalized expression values obtained with the *VIM* long isoform-specific primers showed to be significantly higher in WB (2.45 ± 0.35) and SM samples (2.58 ± 0.33) compared to NORM group (1.60 ± 0.44; *P* ≤ 0.01 for WB vs. NORM and *P* ≤ 0.01 for SM vs. NORM comparison; Figure 4A). Group effect was also found significant for the transcription levels of the *VIM* sequence common to both *VIM* isoforms (*P* ≤ 0.05; Table 1) but with higher intra-group variability. Therefore, although the SM mean for *VIM* common sequence (5.48 ± 5.58) was four times higher than that of NORM group (1.31 ± 0.86), due to the high intra-group standard deviations, no significant (*P* > 0.05) differences were found and only a trend toward significance between NORM (1.31 ± 0.86) and WB groups (4.37 ± 3.36; *P* = 0.08; Figure 4B) was observed. The *DES* gene expression showed a significant increase when passing from NORM to WB group (0.37 ± 0.23 and 1.01 ± 0.59 in Table 1, respectively) and a decrease in the *DES* normalized mean for SM samples (0.85 ± 0.40, Table 1). Both WB and SM groups significantly differed from NORM samples (*P* = 0.05 for both WB vs. NORM and SM vs. NORM comparisons, Figure 4C), showing anyway a consistent intra-group variability. The normalized transcription levels of the *DES* gene resulted to be positively correlated with *VIM* common sequence expression both when correlations are performed considering all the studied samples together or each group separately (Table 2). Conversely, a positive correlation (*r* = 0.90; *P* ≤ 0.05) was noticed between *DES* and *VIM* long-isoform in NORM samples, but the same genes yielded an opposite correlation in the WS group (*r* = −0.99; *P* ≤ 0.001). Interestingly, also the two studied VIM sequences followed a similar trend, with a positive correlation linking them in NORM individuals (*r* = 0.900; *P* ≤ 0.05) that became negative in WS ones (*r* = −0.99; *P* ≤ 0.001; Table 2). Similarly to what was observed for proteins, the slow-growing genotype showed considerably lower normalized expressions for both *VIM* sequences and *DES* compared to the NORM group. For *VIM* long isoform-specific sequence, individuals belonging to the slow-growing breed showed a normalized transcription level 15-times lower than the NORM group (0.10 ± 0.02 in slow-growing genotype; *P* ≤ 0.01 for the difference). Notably lower expressions were also noticed for *VIM* common sequence (0.07 ± 0.01) and *DES* (0.10 ± 0.04).

**TABLE 1:** One-way ANOVA results for the type of abnormality effect.

**Gene**	***N***	**Mean**	***SD***	**Group effect**	
				***F*-value**	**P(>F)**
*VIM* (long transcript)				14.89	0.001
NORM	5	1.60	0.44		
WS	5	1.99	0.64		
WB	5	2.45	0.35		
SM	5	2.58	0.33		
All	20	2.16	0.58		
*VIM* (common sequence)				4.58	0.046
NORM	5	1.31	0.86		
WS	5	2.51	2.71		
WB	5	4.37	3.36		
SM	5	5.48	5.58		
All	20	3.42	3.66		
*DES*				3.45	0.079
NORM	5	0.37	0.23		
WS	5	0.77	0.51		
WB	5	1.01	0.59		
SM	5	0.85	0.40		
All	20	0.75	0.48		

*For each group, mean and standard deviation (SD) of the normalized gene expressions are reported.*

**TABLE 2:** Spearman’s correlations between the expression levels of *VIM* and *DES* genes considering all the samples and each type of abnormality.

**All**	***VIM***	***VIM***	***DES***
	**(long transcript)**	**(common sequence)**	
*VIM*(long transcript)	1	n.s.	n.s.
*VIM*(common sequence)	n.s.	1	**0.90^∗∗∗^**
*DES*	n.s.	**0.90^∗∗∗^**	1

	**NORM**		
**SM**			
*VIM*(long transcript)	1	**0.90^∗^**	n.s.
*VIM*(common sequence)	n.s.	1	**0.90^∗^**
*DES*	n.s.	**0.90^∗^**	1

	**WS**		
**WB**			
*VIM*(long transcript)	1	**−0.99^∗∗∗^**	**−0.99^∗∗∗^**
*VIM*(common sequence)	n.s.	1	**0.99^∗∗∗^**
*DES*	n.s.	**0.90^∗^**	1

*Significant correlations are reported in bold. ^∗^*P* ≤ 0.05; ^∗∗∗^*P* ≤ 0.0001. n.s., not significant.*

## Discussion

In the present study, the distribution and expression of DES and VIM have been assessed on fast-growing having either macroscopically normal appearance (NORM) or affected by muscular abnormalities, namely WS, WB, and SM. Overall, the results obtained through immunohistochemistry evidenced the presence of fibers immunoreactive to VIM as well as positively stained to both VIM and DES in all the experimental groups. Since VIM and DES are normally expressed during muscle regeneration and their localization was proposed as a reliable marker of muscle fiber regeneration (Bornemann and Schmalbruch, 1992; Gallanti et al., 1992; Yang and Makita, 1996), it might be assumed that those fibers immunoreactive to VIM and/or positive for both VIM and DES were undergoing regenerative processes. However, some fibers showed immunoreactivity to VIM but not to DES. It can thus be likely hypothesized that different consecutive phases of regeneration were taking place within these muscles as also evidenced by the small diameter of these fibers themselves. Indeed, VIM is mainly expressed in immature myotubes and myoblasts (Price and Sanger, 1982) and its expression gradually decreases as their development proceeds (Tokuyasu et al., 1985). In this context, the fibers immunoreactive to VIM might be considered at the beginning of the regenerative process in which this protein is transiently expressed (Gallanti et al., 1992). On the other hand, it might be hypothesized that in those fibers positively stained to both VIM and DES an advanced phase of muscle regeneration is in progress thus leading to a strong immunoreactivity to DES (Bornemann and Schmalbruch, 1992; Gallanti et al., 1992). In detail, the presence of fibers immunoreactive to both the intermediate filaments is particularly evident in those muscles affected by abnormalities (WS, WB, and SM). These results are in agreement with the findings of previous studies carried out on dystrophic muscles in which the expression and distribution of DES and VIM have been assessed (Cooper et al., 1988; Gallanti et al., 1992; Holland et al., 2015; Fröhlich et al., 2016). Indeed, an increased expression of VIM and DES was considered as a reliable marker of muscle regeneration (Bornemann and Schmalbruch, 1992; Gallanti et al., 1992; Fröhlich et al., 2016) and hypothesized to be a compensatory mechanism supporting the structural organization of the sarcomere (Gallanti et al., 1992). Besides, if compared with NORM muscles belonging to the fast-growing genotype, the few fibers immunoreactive to VIM and DES observed in the slow-growing genotype, suggest that in these animals, not selected for growth performances, only mild regeneration processes, ascribable to a physiological turnover, occur.

The results of Western Blot analyses corroborate those obtained through immunohistochemistry and provide elements to further characterize the muscles exhibiting WS, WB, and SM. In detail, the significantly higher amount of VIM observed in WB suggests the development of intense regenerative processes in an early-stage within these muscles. On the other hand, the 1.5-fold increase in DES content found in WS might be attributed to a progression of the regenerative processes that require the synthesis of this intermediate filament protein to preserve the structural organization of the developing fibers. Therefore, overall, it might be speculated that regenerative processes were taking place in both WB and WS muscles even though a different progression step in the development of regenerative fibers was observed. This hypothesis could be further tested in follow-up investigation, by measuring the expression of VIM and DES at different stages of the animals’ growth. Also, the significantly lower content of VIM and DES observed in the muscles belonging to the slow-growing genotype (29.4 and 49.3%, respectively), in comparison with their NORM fast-growing counterpart, confirmed the occurrence of a less intense regeneration that might be barely related to a physiological turnover of the muscle fibers. It might be pointed out that our immunoblots for VIM and DES revealed the presence of a single band that, according to the marker, has a molecular weight of approximately 110 kDa. These results are in agreement with the findings obtained in previous studies (Traub et al., 1993; Henderson et al., 2017). Thus, considering the high specificity of the antibodies used for Western Blotting (the same adopted for immunohistochemistry), we hypothesized that this band might be the result of the formation of dimers, either homodimers (only VIM or DES) or heterodimers (VIM-DES) at the Z-disk (Henderson et al., 2017). Indeed, in various developmental and pathological conditions, VIM and DES are co-expressed (Stewart, 1990; Traub et al., 1993) and were observed to form heterodimers (Traub et al., 1993; Henderson et al., 2017).

About gene expression results, both couples of primers designed on the *VIM* gene successfully amplified all samples and the two isoforms reported in the Ensembl database for *VIM* gene were quantified. Therefore, these results suggest that also a longer *VIM* isoform is commonly expressed in the *P. major* muscle of fast-growing. This longer *VIM* isoform (Ensembl ID = ENSGALT00000014123.6) is reported to have a longer promoter and exon 1 sequences compared to the shorter *VIM* transcript (Ensembl ID = ENSGALT00000083582.2; last accessed on 30^th^ of January 2019). Similar alternative usage of the gene promoter and first exon has been also observed for human *VIM* gene in breast cancer and adrenal carcinoma cell lines (Zhou et al., 2010), but the functional properties of these transcripts are still unclear. In the present study, both *VIM* common sequence and *VIM* long transcript-specific sequence showed a concurrent expression in the samples, in agreement with the results in human cells (Zhou et al., 2010), but different patterns of correlations were noticed between the two *VIM* sequences as well as between them and *DES* gene expression. In particular, despite both *VIM* sequences followed a similar increasing trend when passing from NORM to SM groups, the two isoforms were positively correlated in broilers exhibiting a macroscopically normal appearance, while the same were negatively correlated in the *P. major* muscles affected by WS condition. This twist in the correlations can suggest that the splicing of the alternative *VIM* transcripts may have a role in the occurrence of WS abnormality and that, more in general, a co-regulation pattern may exist between the two *VIM* transcripts. This hypothesis would need anyway to be proven with further dedicated studies, to elucidate the possible effects of the different *VIM* transcripts in macroscopically normal and in abnormal *P. major* muscles. Moreover, the two *VIM* sequences showed opposite correlations with *DES* gene expression in the WS group, suggesting that the two *VIM* isoforms may also play distinct functional roles in the regenerative process taking place in WS affected muscles. On the whole, these results seem to confirm that WS muscles may be in a more advanced stage of muscle regeneration, since the negative correlation occurring between *DES* and *VIM* long transcript-specific sequence reflects the negative relation linking *VIM* and *DES* during the development of immature myotubes, where *DES* expression gradually increases while *VIM* is down-regulated (Sejersen and Lendahl, 1993).

The comparison between the expression of the *VIM* and *DES* genes and the protein levels further evidenced that, although the highest level of expression of *DES* gene was found in WB, this is not associated with a higher amount of DES protein, whose content was found to be significantly higher in WS. We hypothesize that this might be attributed to a potential delay in the DES protein translation as a consequence of the remarkably higher VIM content observed in WB muscles. Indeed, a higher VIM content was found in WB and suggested that the higher *VIM* gene expression results in a successfully translated protein. These results seem to support the hypothesis that a more advanced regeneration can be observed in WS in comparison with WB in which regenerative processes in an early-stage associated with still high levels of translated VIM protein are found. Intriguingly, no linear correspondence between *VIM* gene expression and its protein product was observed in SM affected muscles. Indeed, the highest *VIM* mRNA level in these muscles was associated with a significantly lower content of the relative protein. This unexpected result together with the strong VIM immunoreactivity observed in fibroblasts, in agreement with previous studies (Bornemann and Schmalbruch, 1992; Gallanti et al., 1992; Chang et al., 2002), led us to consider in deeper the mechanisms underlying *VIM* transcription and its translation processes. In detail, this evidence observed also in our samples, may reflect the regulative action exerted by VIM on fibroblast proliferation and differentiation (Cheng et al., 2016). VIM has been proven to coordinate fibroblast proliferation in regenerative processes as a response to wound healing (Cheng et al., 2016). Thus, this suggests that VIM protein plays a major role in the regulation of pathological fibrosis in which differentiated fibroblasts are responsible for inducing fibrosis via increased extracellular matrix synthesis and impedes normal function of the organ (reviewed in Darby and Hewitson, 2007). This role of VIM in regulating fibroblasts proliferation (Cheng et al., 2016) seems to be consistent with the results obtained in the present research in which the highest VIM content is observed in WB affected muscles that concurrently exhibit proliferation of connective tissue. Within this context, the increased expression of *VIM* together with the higher content of the related protein in WB may have induced the fibro-adipogenic precursors to differentiate into fibroblasts. On the other hand, the lack of translation of *VIM* mRNA into VIM protein noticed in SM samples may have resulted in an impaired differentiation of fibro-adipogenic precursors to fibroblasts (as evidenced by the findings of immunohistochemical analyses). It can be likely speculated that this may lead to an altered extracellular matrix synthesis that inducing a reduction in the number of fibroblasts within the perimysial compartment, ultimately, results in the progressive rarefaction of the perimysial connective tissue that is distinctively observed in SM affected muscles. However, this hypothesis needs to be tested in future studies. Interestingly, the amount of VIM protein has been already reported to depend mainly on post-transcriptional regulation of *VIM* gene expression (Beggs et al., 2015), thus suggesting that a similar regulative process may have occurred also in SM samples. Therefore, we hypothesize that the different phenotypes associated with WS, WB, and SM in *P. major* muscles may be at least in part, a direct consequence of VIM protein synthesis. Moreover, a deeper knowledge of the molecular processes influencing both the VIM protein synthesis from mRNA and the post-transcriptional regulation of gene expression is needed to assist the identification of the factors involved in the occurrence of the muscular growth-related abnormalities in broilers.

## Conclusion

The distribution and expression of the intermediate filament VIM and DES proteins can be considered a reliable marker of the regenerative processes taking place within the *P. major* in modern broilers affected by WS, WB, and SM abnormalities but also in those having a macroscopically normal appearance. Overall, the patterns of immunoreactivity together with the results obtained through immunoblotting and gene expression suggested that intense regenerative processes were taking place in both WB and WS muscles even though a different progression in the development of regenerative-fibers can be likely hypothesized, with the first being in an early stage of regeneration if compared with WS once. This could be further tested in follow-up investigation, by measuring the expression of VIM and DES at different stages of the animals’ growth. On the other hand, the lack of correspondence between *VIM* gene expression and its protein product observed in SM suggested that *VIM* may also have a major role in the occurrence of SM phenotype. Anyway, further research will be needed to understand if post-transcriptional mechanisms affecting the translation of *VIM* mRNA to protein can exert an important effect in the development of the WB and SM abnormalities.

## Data Availability Statement

The raw data supporting the conclusions of this article will be made available by the authors, without undue reservation, to any qualified researcher.

## Ethics Statement

All the aspects related to farming, handling, transportation, and slaughtering of the birds strictly accomplished with the European Union legislation.

## Author Contributions

All authors listed have made a substantial, direct and intellectual contribution to the work, and approved it for publication. MP, FSi, and FSo planned the experiment. MP, FSo, MM, FSi, and RD designed the study. FSo, MM, MZ, MD, EB, and MB performed the laboratory analyses. FSo, MD, and EB performed Western blot and SDS page analysis and interpreted the results. MM and PC performed the immunohistochemical analysis. MZ and MB performed the gene expression quantitation. FSo, MZ, and MB organized the databases and performed the statistical analysis. FSo, MZ, MP, RD, and MM wrote the first draft of the manuscript. All authors contributed to manuscript revision.

## Conflict of Interest

The authors declare that the research was conducted in the absence of any commercial or financial relationships that could be construed as a potential conflict of interest.

## Footnotes

1 http://www.bioinformatics.nl/cgi-bin/primer3plus/primer3plus.cgi
