# Supplementary material for: Distribution and Expression of Vimentin and Desmin in Broiler Pectoralis major Affected by the Growth-Related Muscular Abnormalities
Source: Front Physiol. 2020 Jan 17;10:1581. doi: 10.3389/fphys.2019.01581 (PMC6978684; doi:10.3389/fphys.2019.01581)
Supplement: Supplementary file 1 [file Table_1.DOCX]

Supplementary Table 1. Summary of the primers used for the genes expression quantitation and their annealing temperature ™.

| **Gene name** | **Acronym** | **Accession number** | **Primers** | **Primer location** | **TM (°C)** | **Amplicon Length (bp)** | **Reference** |
| --- | --- | --- | --- | --- | --- | --- | --- |
|  | ***Target genes*** | | | | | | |
| *Vimentin (long transcript)* | *VIM* | ENSGALT00000014123.6 | F: 5’- TGCCTATTCCAACCTTTGCT -3’ | Exon 7 | 60 | 152 |  |
|  |  |  | R: 5’- ATCGTGATGCTGGGAAGTTT-3’ | Exon 9 |  |  |  |
| *Vimentin (common sequence)* | *VIM* | ENSGALT00000014123.6  ENSGALT00000083582.2 | F: 5’- CGCTACATCACGTCCAGCAC -3’ | Exon 1 | 60 | 177 |  |
|  |  |  | R: 5’-GTCCGCCAGGGTGAAGTC -3’ | Exon 1 |  |  |  |
| *Desmin* | *DES* | ENSGALT00000018446.6 | F: 5’-GCGTGACAACCTGCTAGATG-3’ | Exon 1 | 60 | 102 |  |
|  |  |  | R: 5’-GCTCTGAAAGCAGCCAAGTT-3’ | Exon 2 |  |  |  |
|  | ***Housekeeping genes*** | | | | | | |
| *Glyceraldehyde-3-phosphate dehydrogenase* | *GAPDH* | ENSGALT00000086032.2 | F: 5’- TGACAGCCATTCCTCCAC-3’ | Exon 10 | 66 | 126 | Zambonelli et al., 2016 |
|  |  |  | R: 5’- TGGACCATCAAGTCCACAAC -3’ | Exon 11 |  |  |  |
| *Ribosomal Protein L32* | *RPL32* | ENSGALT00000013725.4 | F: 5’- ATGGGAGCAACAAGAAGACG -3’ | Exon 3 | 66 | 139 | Zambonelli et al., 2016 |
|  |  |  | R: 5’- TTGGAAGACACGTTGTGAGC -3’ | Exon 4 |  |  |  |
| *Tyrosine 3-Monooxygenase/Tryptophan*  *5-Monooxygenase Activation Protein Zeta* | *YWHAZ* | ENSGALT00000087563.2 | F: 5’- TTGCTGCTGGAGATGACAAG -3’ | Exon 3 | 66 | 60 | Zambonelli et al., 2016 |
|  |  |  | R: 5’- CTTCTTGATACGCCTGTTG -3’ | Exon 4 |  |  |  |
